# Liquefied Lung Cancer: An Uncommon Form of Squamous Cell Carcinoma of the Lung

**DOI:** 10.7759/cureus.41848

**Published:** 2023-07-13

**Authors:** Nismat Javed, Somin Lee, Srikaran Bojja, Umesh Tiwari, Misbahuddin Khaja

**Affiliations:** 1 Internal Medicine, BronxCare Health System, Icahn School of Medicine at Mount Sinai, New York, USA; 2 Internal Medicine/Pulmonary Critical Care, BronxCare Health System, Icahn School of Medicine at Mount Sinai, New York, USA

**Keywords:** diagnosis, management, liquefication, necrosis, squamous cell carcinoma of the lung

## Abstract

Lung cancer is a significant global health concern, with high incidence and mortality rates. This case report presents the atypical presentation of a 71-year-old female with a history of lung cancer who initially presented with symptoms suggestive of infection secondary to a liquefied lung malignancy and later developed bronchial obstruction. Diagnosis of lung cancer requires a high level of clinical suspicion, and imaging techniques, such as PET and CT scans, provide additional evidence. However, necrotic lesions do not have specific findings on radiology. Treatment options depend on the cancer stage, with surgical resection being the primary approach. Chemotherapy and radiation are used for unresectable cases. Liquefied lung cancer is associated with poor outcomes. Post-obstructive pneumonia with necrotic lesions, particularly in cases without an underlying organism, is a relatively rare phenomenon in lung cancer that requires further investigation. Large-scale studies are needed to explore this aspect further and enhance our understanding of lung cancer complications.

## Introduction

Lung cancer remains a cause of global concern. Estimates reveal approximately 250,000 to 400,000 new cases of lung cancer being diagnosed in women [1]. In fact, lung cancer is the second most common malignancy being diagnosed globally and remains the leading cause of deaths worldwide [1]. In the US, about 654,620 men and women have been diagnosed with lung cancer in the past five years, and estimates reveal additional 236,740 cases to be diagnosed by 2022 [2]. Clinical presentation can vary from asymptomatic state to hemoptysis, pleural effusion, and weight loss [3]. Necrosis in lung cancer is usually caused by vascular involvement of tumor cells and bronchial obstruction leading to ischemia, which increases the likelihood of a poor prognosis. It is significantly observed in squamous cell carcinomas [4]. Cavitary lesions can appear as bubble-like lesions in adenocarcinoma that might comprise of a small ectatic bronchus or thin-walled lesions in squamous cell carcinoma [4]. Here, we present the case of a 71-year-old female with a history of lung cancer who presented with symptoms suggestive of infection owing to liquefied lung malignancy and was later diagnosed with bronchial obstruction.

## Case presentation

A 71-year-old female patient presented to our institution's emergency department with chief complaints of progressive shortness of breath, associated with a productive cough. The patient had dyspnea on minimal exertion for the past few weeks, but her shortness of breath progressively worsened and disrupted her daily activities. Upon emergency department (ED) arrival, the patient was hypoxic (SpO_2_: 83%) on room air and placed on high-flow nasal canula therapy for oxygen supplement. Other vital signs showed sinus tachycardia of 105 beats per minute, blood pressure of 145/63, afebrile to 98.9 F, and tachypnea respiratory rate of 22 per minute. On physical examination, the patient was alert and oriented but appeared tired and had bilateral decreased air entry with diffuse wheezing and rhonchi.

**Figure 1:**
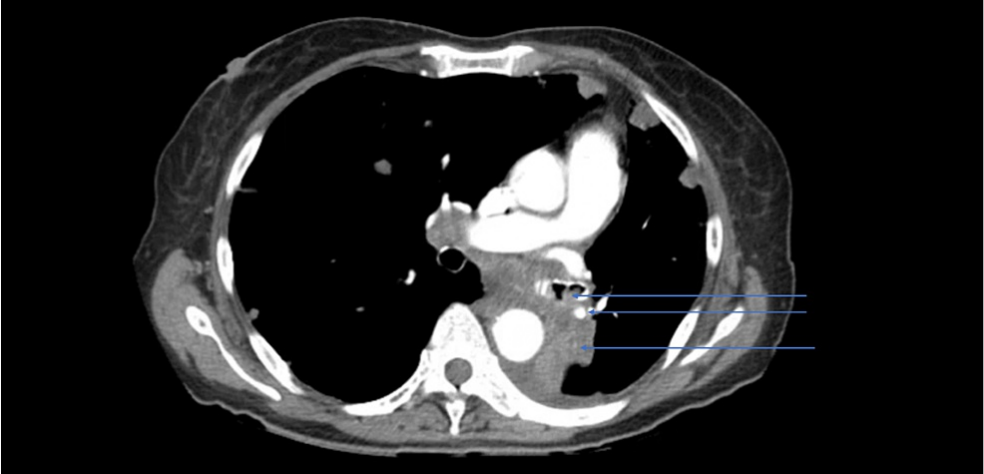
CT scan of the chest showing adenopathy and soft tissue in the left bronchus (blue arrows illustrate the findings)

Her past medical history was significant for stage IV squamous cell carcinoma of the lung, bulky left lower lobe primary malignancy, with left mainstem multiple endobronchial lesions, T3 N0 M1a, stage IV disease on chemotherapy with docetaxel and ramucirumab every three weeks, and other medical comorbidities, including hypertension, chronic obstructive pulmonary disease (COPD), hypothyroidism, type II diabetes mellitus, chronic hepatitis C, anemia, and peripheral vascular disease. Her social history revealed that she was a former smoker and had a 50-year smoking history. Due to her elevated risk of malignant-related thrombophilic status, a CT scan of the chest (Figure 1) was performed, and pulmonary embolism was ruled out.

However, complete collapse and consolidation of the left lower lobe are related to the obstruction of the left lower lobe bronchus by mass and bilateral numerous pulmonary masses. 

**Figure 2:**
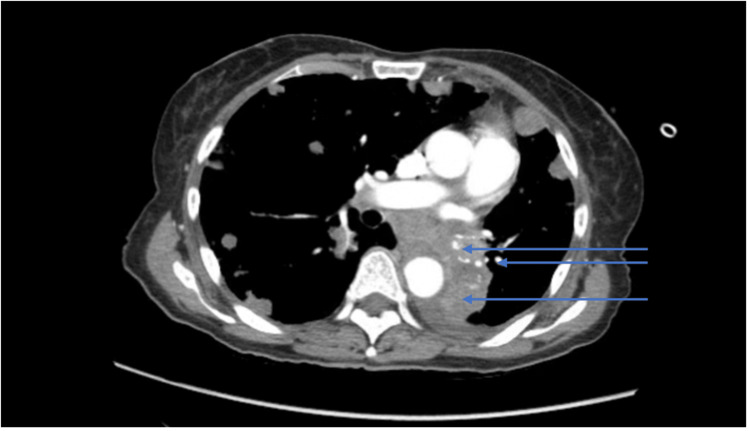
CT scan of the chest showing increase in pulmonary masses and consolidations (blue arrows illustrate the findings)

Labs were significant for leukocytosis of 14,100/ul, with left shift, neutrophil of 81.9%, anemia (hemoglobin 10.0 g/dl), reactive thrombocytosis of 468,000/ul, elevated D-dimer of 712 ng/ml, hypercalcemia of 10.8 mg/dl, and elevated lactic acid of 1.7 mmol/L. The patient was admitted to the medicine-pulmonary floor for post-obstructive pneumonia and started on empiric antibiotics, azithromycin, piperacillin-tazobactam, and vancomycin. However, the patient had worsening leukocytosis and persistent fever despite empiric antibiotic coverage for pneumonia. Four weeks later, repeat CT chest (Figure 2) showed an increase in the sizes of the bilateral pulmonary masses compared to the prior initial CT scan.

There was a concern that either those multiple pulmonary lesions are metastatic or infectious due to their fast growth. As the patient was on active chemotherapy prior, her immunocompromised status in the setting of malignancy would make her susceptible to other opportunistic infections. 

**Figure 3:**
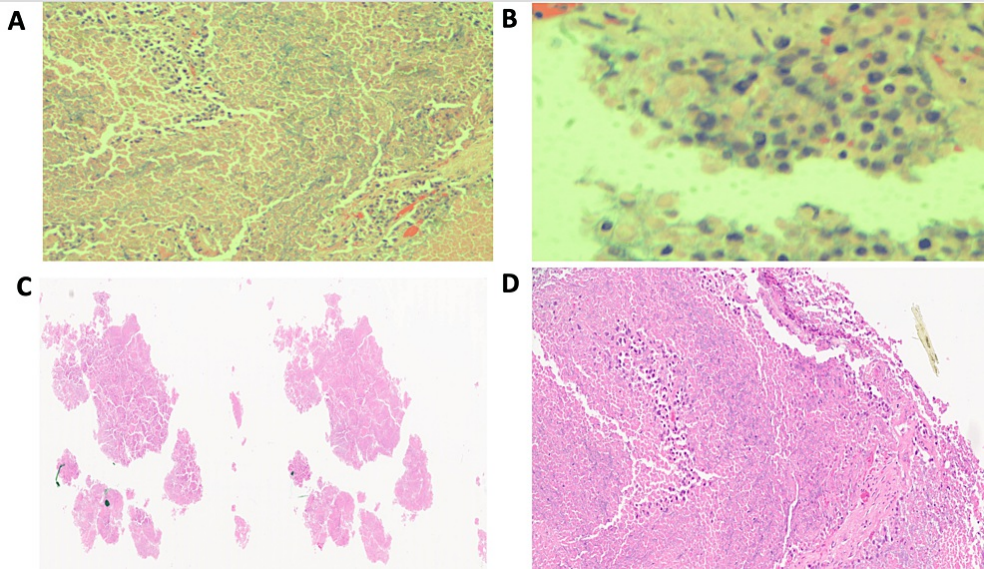
Pathology results Extensive necrosis with rare aggregates and detached epithelial cells observed on low (A,C) and higher magnification (B,D). The relatively well preserved cells have dense cytoplasm and atypical hyperchromatic nuclei as seen in Figure A-D. Figure credits: Misbahuddin Khaja

Infectious diseases were consulted, and infectious workups were negative for *Legionella* antigen, histoplasma antigen, *Aspergillus* antigen, *Coccidiomycosis* antigen, cryptococcal antigen, and 1-beta-D-glucan. Mycobacterial cultures and sputum for acid-fast bacilli were also negative. The oncology team was consulted and recommended a biopsy of multiple pulmonary lesions to rule out any superimposed infection. Interventional radiology performed a biopsy of multiple pulmonary lesions as bronchoscopy was deemed high risk for the patient. Gross examination of the pathology specimen revealed a cystic lesion with liquefied material. Analysis from the aspirate revealed WBC count of 183 cells/mm^3^, 88% neutrophils, pH 6.5, lactate dehydrogenase (LDH) of 721 units/L, protein of 0.2 g/dl, and glucose of 2 mg/dl, consistent with exudate. Aerobe and anaerobe cultures had no growth of any bacteria. Cytology reported extensive necrotic samples. Pathology reported highly atypical degenerating epithelial cells, suggestive of squamous cell carcinoma. Figure 3 details the pathology results. Atypical cells stained positive for CK5 and p40 and negative for thyroid transcription factor 1 (TTF-1), and napsin-A on immunohistochemistry. 

The patient was transferred to a tertiary center for possible distal left main bronchus stent, with mechanical tumor debridement to alleviate symptoms for palliative purposes. On bronchoscopy, an occluded left lower lobe with a significant amount of endobronchial and submucosal disease resulting in obstruction was noted. Balloon dilation was performed in the left upper lobe and mechanical debridement of the tumor, but no significant improvement was appreciated in the distal left lower lobe of the airway lumen. A bronchial stent was unable to place. She received two days of high-dose radiation therapy, but the patient refused to continue the treatment. The goal of care was discussed through palliative medicine. 

The hospital stay was complicated by persistent worsening hypercalcemia secondary to malignancy. Hypercalcemia was treated with intravenous hydration and zoledronic acid. However, her clinical course progressively worsened with metabolic encephalopathy, abdominal pain, diarrhea, dysphagia, and decreased appetite. With a progressive decline in her overall status and complications, the palliative team was followed. She was, therefore, discharged to inpatient hospice care.

## Discussion

Lung cancer is a major cause of death among men and women worldwide. The average age of diagnosis is 70 years old, and men are twice as likely as women to be diagnosed with lung cancer, owing to differences in tobacco consumption [5]. Risk factors for development of lung cancer include African American race, smoking, second-hand smoking, radon exposure, occasional exposure to carcinogens, family history, infections including* Mycobacterium tuberculosis*, and, to a lesser extent, hormonal issues and diabetes mellitus [5,6]. The patient’s age was relatively similar to previously reported epidemiological characteristics. In addition, she had a history of COPD, smoking, and diabetes mellitus before being diagnosed with lung cancer. 

Presenting features include cough, hemoptysis, pleural chest pain, and additional symptoms suggestive of paraneoplastic syndromes [7]. Cough and dyspnea were also suggestive of post-obstructive pneumonia in exophytic lesions [7]. Chest pain also implied pleural involvement in the form of pleural effusion or metastasis [7]. Labs are relatively nonspecific with patients having anemia or thrombocytosis because of underlying malignancies [2]. These features were present in the case presented here. 

Post-obstructive pneumonia can also be one of the presenting features. The presence of advanced lung cancer, specifically in patients with a diagnosis of squamous cell carcinoma and hilar involvement, is commonly associated with the presence of post-obstructive pneumonia [8]. Usually, *Streptococcus pneumoniae*,* Bacillus cereus*,* Haemophilus influenzae*, *Stenotrophomonas maltophilia,* and *Escherichia coli* have been observed in microorganism identification in patients with post-obstructive pneumonia [9]. There is limited evidence of patients with post-obstructive pneumonia and persistent fevers without any underlying organism, as observed in our case. In addition, cavitary lesions on imaging were commonly associated with post-obstructive pneumonia [8]. 

Squamous cell carcinoma of the lung is commonly associated with central cavitation and necrosis [4,10]. It has been hypothesized that cavity formation occurs due to rapid tumor growth that overwhelms the supporting blood supply of the tumor, resulting in necrosis. This process has been implied as a source of infection but not in cases of post-obstructive pneumonia as observed in our patient. While the feature did signify shorter disease-free interval and poor response to therapy, bronchial obstruction following the development of such features is rare [10]. Antibiotic treatment was relatively similar to normal infectious regimens and focused on targeting microbial etiologies and polymicrobial flora [9]. In patients with refractory post-obstructive pneumonia, a relatively graver disease course was present with patients dying after 30 days of diagnosis [9]. Squamous cell carcinoma was more frequently associated with necrosis as compared to adenocarcinoma in a previous study. The proposed mechanism, known as a check valve, is caused by infiltration of tumor cells into the bronchiolar lumen that results in elastic retraction. Moreover, due to the similar mechanism, air bronchograms are less observed in squamous cell carcinoma [4].

Diagnosis requires high clinical suspicion. Imaging, including PET and CT scans, provide additional evidence. Invasive imaging might be indicated in patients with nodal and endobronchial disease as observed in our patient [11]. While necrosis might be visualized on CT scan, the diagnostic value is limited without a prior history because of the vast differential diagnosis [4].

The treatment varies with the stage of cancer. Surgical resection is usually the mainstay of treatment. In the case of unresectable cancer, chemotherapy and radiation are used [11]. Necroptosis, as observed in this patient, has a role in anti-cancer therapy. Triggering caspase-independent cell death, for example, via etoposide, 5-fluorouracil (5-FU), and cisplatin, forms the basis of cancer therapeutics [12]. However, more studies are needed to determine the application of these specific therapeutics in the cohort with extensive necrosis.

Complications can include symptomatic hypercalcemia, as observed in our case, syndrome of inappropriate antidiuretic hormone secretion (SIADH), neurologic paraneoplastic syndromes, hypertrophic pulmonary osteoarthropathy, dermatomyositis, and polymyositis [7] that are associated with a poor prognosis. Endobronchial obstruction and superior vena cava syndrome might also be present and require additional interventional procedures [7,11].

## Conclusions

Lung cancer has far-reaching global consequences and plays an important role in burdening healthcare. Post-obstructive pneumonia with a necrotic lesion as one of the complications of lung cancer is a relatively less common feature that is yet to be explored, specifically in patient cohorts without an underlying organism. Further studies are warranted to explore the phenomenon on a larger scale.
